# Editorial: Machine learning methods for human brain imaging

**DOI:** 10.3389/fninf.2023.1154835

**Published:** 2023-02-28

**Authors:** Fatos Tunay Yarman Vural, Sharlene D. Newman, Tolga Çukur, Itır Önal Ertugrul

**Affiliations:** ^1^Department of Computer Engineering, Middle East Technical University, Ankara, Turkey; ^2^Alabama Life Research Institute, The University of Alabama, Tuscaloosa, IN, United States; ^3^Department of Electrical and Electronics Engineering, Bilkent University, Ankara, Turkey; ^4^Department of Information and Computing Sciences, Utrecht University, Utrecht, Netherlands

**Keywords:** machine learning, deep learning, artificial intelligence, imaging, MRI

The use of artificial intelligence (AI) methods like machine learning (ML), including deep learning, to make sense of brain imaging data has exploded over the past 10 years. Some of the early work focused on classifying brain states measured with functional magnetic resonance imaging (Mitchell et al., 2004). Those studies were exciting and demonstrated the potential power of ML to classify brain states in a way that reveals something about human cognition. ML is used in multiple aspects of brain imaging including image acquisition, reconstruction, analysis, and reporting (Aggarwal et al., 2023). For example, there are numerous studies using ML to classify groups of patients to improve diagnosis of neurodevelopmental disorders (e.g., autism, Parlett-Pelleriti et al., 2022), psychological disorders (e.g., schizophrenia, Chilla et al., 2022; and depression, Bhadra and Kumar, 2022), the progression of dementia (Mirzaei and Adeli, 2022) and tumors (Soomro et al., 2022), among others. On the image analysis side, ML applications are numerous and include it being used to improve denoising of image data (Gregory et al., 2021) and image segmentation (Wang et al., 2020).

The Research Topic, “*Machine learning methods for human brain imaging*,” is a small sampling of 11 research articles that demonstrate the use of ML in multiple contexts and with multiple imaging modalities. The Research Topic includes two manuscripts (Alchihabi et al.; Fang et al.) that take different approaches to understanding cognitive networks—one using multi-variate pattern dependencies between brain regions and another examining network dynamics during the execution of a task. There are also three studies designed to use AI to diagnose psychological disorders—one using MRI to diagnosis defiant disorders in children (Menon and Krishnamurthy), one using EEG to classify brain states in schizophrenia patients and healthy controls (Plechawska-Wójcik et al.) and another classifying patients with obsessive-compulsive disorder and controls (Luo et al.). A third group of studies use ML to address analytic issues including one developing an open access tool for whole brain segmentation (Manjón et al.) and volumetric analysis of large datasets, one using fuzzy neural networks to improve 2D to 3D image transformations (Tavoosi et al.), and registration of multimodal 2D coronal section images of gene expressions in the mouse brain (Krepl et al.).

One goal of AI is to create systems that function like the human brain (Hopgood, 2005). Current systems fall short and two of the manuscripts in this Research Topic attempt to address this issue (Matsui et al.; Zhang et al.). Deep learning systems, for example do a great job of mimicking human vision, to a point; their mapping from stimulus input to perceptual output are different with respect to adversarial images. Zhang et al. attempts to characterize the differences in how AI systems and human brains process these adversarial images by comparing artificial neural networks and human brain activation, using what is learned to improve AI performance.

The use of ML in human brain imaging is only expected to increase. The power of deep learning methods makes them attractive for analyzing the growing number of large, publicly available datasets. However, it is important to slow down to evaluate their efficacy as well as to evaluate their weaknesses. One such weakness is addressed in the manuscript by Varotto et al.—how to handle imbalanced datasets. Most large datasets do not have even distributions of minority populations (e.g., racial, socioeconomic, patient, etc.). This is only one such shortcoming that demonstrates the need for careful evaluation.

## Author contributions

All authors listed have made a substantial, direct, and intellectual contribution to the work and approved it for publication.

## References

[B1] Aggarwal K. Jimeno M. M. Ravi K. S. Gonzalez G. Geethanath S. (2023). Developing and deploying deep learning models in brain MRI: a review. arXiv preprint arXiv:2301.01241. 10.48550/arXiv.2301.01241 37539775

[B2] Bhadra S. Kumar C. J. (2022). An insight into diagnosis of depression using machine learning techniques: a systematic review. Curr. Med. Res. Opin. 38, 749–771. 10.1080/03007995.2022.2038487 35129401

[B3] Chilla G. S. Yeow L. Y. Chew Q. H. Sim K. Prakash K. B. (2022). Machine learning classification of schizophrenia patients and healthy controls using diverse neuroanatomical markers and Ensemble methods. Sci. Rep. 12, 2755. 10.1038/s41598-022-06651-4 35177708 PMC8854385

[B4] Gregory S. Cheng H. Newman S. Gan Y. (2021). HydraNet: a multi-branch convolutional neural network architecture for MRI denoising, in Medical Imaging 2021: Image Processing, Vol. 11596 (Washington, DC: SPIE), 881–889. 10.1117/12.2582286

[B5] Hopgood A. A. (2005). The state of artificial intelligence. Adv. Comput. 65, 1–75. 10.1016/S0065-2458(05)65001-2

[B6] Mirzaei G. Adeli H. (2022). Machine learning techniques for diagnosis of Alzheimer disease, mild cognitive disorder, and other types of dementia. Biomed. Signal Process. Control 72, 103293. 10.1016/j.bspc.2021.103293

[B7] Mitchell T. M. Hutchinson R. Niculescu R. S. Pereira F. Wang X. Just M.. (2004). Learning to decode cognitive states from brain images. Mach. Learn. 57, 145–175. 10.1023/B:MACH.0000035475.85309.1b

[B8] Parlett-Pelleriti C. M. Stevens E. Dixon D. Linstead E. J. (2022). Applications of unsupervised machine learning in autism spectrum disorder research: a review. Rev. J. Autism Dev. Disord. 1–16. 10.1007/s40489-021-00299-y

[B9] Soomro T. A. Zheng L. Afifi A. J. Ali A. Soomro S. Yin M.. (2022). Image segmentation for MR brain tumor detection using machine learning: a review. IEEE Rev. Biomed. Eng. 16, 70–90. 10.1109/RBME.2022.3185292 35737636

[B10] Wang J. Cheng H. Newman S. D. (2020). Sparse representation of DWI images for fully automated brain tissue segmentation. J. Neurosci. Methods 343, 108828. 10.1016/j.jneumeth.2020.108828 32603811

